# Prognostic nomogram for post-surgical treatment with adjuvant TACE in hepatitis B virus-related hepatocellular carcinoma

**DOI:** 10.18632/oncotarget.11078

**Published:** 2016-08-05

**Authors:** Hao Hu, Xi Kun Han, Xiao Ran Long, Jia Fan, Zhi Ping Yan, Jian Hua Wang, Rong Liu

**Affiliations:** ^1^ Department of Interventional Radiology, Zhongshan Hospital, Shanghai Medical College, Fudan University, Shanghai, China; ^2^ State Key Laboratory of Cardiovascular Disease, Fuwai Hospital, National Center of Cardiovascular Diseases, Chinese Academy of Medical Sciences and Peking Union Medical College, Beijing, China; ^3^ Sun Yat-sen University Cancer Center, Guangzhou, China; ^4^ Liver Cancer Institute, Zhongshan Hospital, Shanghai Medical College, Fudan University, Shanghai, China

**Keywords:** nomogram, adjuvant transarterial chemoembolization, hepatocellular carcinoma

## Abstract

**Objective:**

This study sought to establish an effective and reliable prognostic nomogram to guide the decision for post-surgical adjuvant transarterial chemoembolization (PA-TACE) in patients with hepatitis B virus-related (HBV) hepatocellular carcinoma (HCC).

**Results:**

The 1, 3, 5-year overall survival rates were, respectively, 87.7%, 52.1% and 28.3% in the patients from the derivation set and 91.7%, 57.1% and 34.1% in those from the validation set. Five risk factors (HBV-DNA level, platelet count, vascular invasion, change of Child-Pugh score, and tumor diameter) in the multivariate analysis were significantly associated with prognosis. The statistical nomogram incorporated these five factors achieved good calibration and discriminatory abilities with c-index of 0.75 (95% CI 0.67 to 0.83). The findings were supported by the independent external validation set (c-index, 0.69; 95% CI 0.56 to 0.83). Patients who had a nomogram score of less than 180 was considered to have higher survival benefit from PA-TACE.

**Methods:**

The nomogram was established based on data obtained from a retrospective study on 235 consecutive patients with HBV HCC who received PA-TACE as an initial therapy from 2006 to 2010 in our center. 84 patients who were collected at another institution between 01/2008 and 12/2010 served as an external validation set. The prognostic nomogram was developed based on the data obtained before the PA-TACE procedure. Predictive accuracy and discriminative ability of the nomogram were assessed by concordance index (C-index), calibration curves, and validation set.

**Conclusion:**

The novel nomogram may achieve an optimal prognostic prediction for PA-TACE in HBV-related HCC.

## INTRODUCTION

Hepatocellular carcinoma (HCC) is the most prevalent primary malignant hepatic tumor and is the third leading cause of cancer-related death [1]. Nearly 70% to 90% of HCC cases develop in patients with chronic cirrhosis of the liver, which is often caused by hepatitis B virus (HBV) persistent infection [2].

Curative hepatic resection is the recommended treatment modality for early HCC with a single nodule and normal liver function but without clinically significant portal hypertension (very early or early HCC, BCLC stage A) [3]. Nevertheless, the prognosis for HBV-related HCC after resection is still discouraging due to the potential for residual tumor and the high rate of tumor recurrence, which exceeds 60% at 5 years post-hepatectomy even in patients with small tumors [4, 5].

Transarterial chemoembolization (TACE) is the treatment approach most commonly used for unresectable HCC. Current guidelines including the BCLC staging system recommend TACE as the standard treatment of intermediate-stage HCC [6]. Because the blood supply of HCC is mainly derived from the hepatic artery, injection of chemotherapeutic drugs and embolizing agents can decrease blood flow to the tumor and induce necrosis of tumor tissues at the embolization regions [7]. The effectiveness of post-surgical adjuvant TACE (PA-TACE) has been investigated in clinical centers worldwide. A randomized controlled study (RCT) showed PA-TACE to be beneficial for patients with HCC larger than 5 cm in diameter, multiple nodules, or macroscopic vascular invasion [8]. Similar results were obtained for patients with microvascular invasion [9]. Recently, two large meta-analysis [10–11] demonstrated that PA-TACE resulted in improved survival rates, especially for tumor with vascular invasion or tumor size >5 cm. Therefore, it is reasonable to establish a reliable and easy-to-use model for preoperative selection which kind of patients can benefit from PA-TACE.

Several factors for predicting the treatment effect of adjuvant chemotherapy after resection. Above all, baseline tumor characteristics before hepatectomy have a significant impact on patient prognosis, including serum alpha-fetoprotein (AFP) and albumin, and platelet count has been associated with tumor recurrence and OS in HCC patients [12, 13]. In addition, relevant studies have shown that post-surgical pathological tumor factors such as tumor number/diameter, presence of vascular invasion or capsule and Edmonson-Steiner classification [12–15] are associated with the OS of HCC patients. Furthermore, HBV reactivation is common after hepatectomy in HBV-related HCC patients. Post-operative persistent high viral load was associated with HCC recurrence and resulted in poor prognosis [16]. Finally, as most patients with HCC have extensive liver cirrhosis, post-operative liver function may further worsen after adjuvant TACE and may negatively impact patient prognosis. However, the factors noted above varied to some extent due to the heterogeneity of the study populations; therefore, comprehensive predictions of survival prognosis have been difficult to make.

Due to the lack of a reliable and pragmatic statistical prediction measures, development of a prognostic predictive system that incorporates parameters associated with PA-TACE based on pre-surgical data becomes urgently needed. Currently, nomogram has been considered to be evidence-based, individualized and highly accurate in prognostic estimation and can widely be developed to many tumors [17–19]. When compared to the traditional predictive systems for many tumors, nomogram has been proposed as alternatives or even as new standards. In this study, we construct a clinically novel and reliable prognostic nomogram for patients with hepatitis B-related HCC treated with PA-TACE. Performance of the nomogram was further verified in independently external validation of patients.

## RESULTS

### Clinicopathologic characteristics of patients

**Figure 1:**
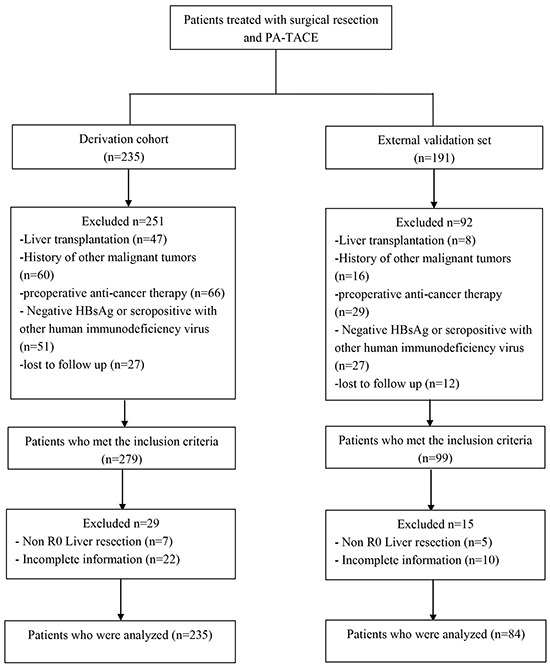
Flowchart of patient selection HBsAg: hepatitis B surface antigen.

A flow chart for derivation and validation cohorts is shown in Figure 1. In the derivation cohort (n=235), the mean age of patients was 52.1 years (SD, 10.2 years), 86.8 % of whom were male. Hepatitis B infection (100 %) is the most common cause of chronic liver disease and approximately 28.5% of enrolled patients were detected as positive HBeAg. A total of 27.7%, 60.9%, and 11.4% of patients were diagnosed at BCLC stage A, B, and C, respectively (n=65, 143, and 27, respectively). The Child-Pugh grade prior to PA-TACE (n=168) increased by at least 1 point compared to that before surgical resection, whereas 67 were unchanged or decreased at least 1 point. In terms of tumor factors, most patients had single tumors (59.1%), and the average diameter of the tumors was 6.5 cm (SD, 4.0 cm). Vascular invasion and capsular infiltration were histologically observed in 125 (53.2%) and 49 (20.9%) patients respectively. Edmondson grade III or IV tumors were noted in 91 (38.7%) patients. Regarding operation factors, 32 (13.6%) required blood transfusion during the perioperative period. No clamping time was observed in 132 (56.2%) patients. Pathological examination revealed cirrhotic livers in most patients. HBV-DNA level reactivation (>10^4^ IU/mL) prior to PA-TACE occurred in 79 (33.6%) patients.

The clinical, histopathological, and surgical factors of the derivation (n=235) and external (n=84) validation sets prior the hepatectomy and PA-TACE are summarized in Table 1. There were no significant differences in baseline characteristics between the derivation and validation set.

**Table 1:** Basal characteristics

Variable	Derivation set (N=235)	Validation set (N=84)	P Value
**Pre-SR**			
Age, y	52.1+-10.2	52.6+-10.7	0.702
Gender			0.762
Male	204 (86.8)	74 (88.1)	
Female	31 (13.2)	10 (11.9)	
HBeAg			0.407
Positive	67 (28.5)	28 (33.4)	
Negative	168 (71.5)	56 (66.6)	
Child-Pugh stage			0.951
A	232 (98.7)	83 (98.8)	
B	3 (1.3)	1 (1.2)	
BCLC-stage			0.977
A	65 (27.7)	24 (28.6)	
B	143 (60.9)	50 (59.5)	
C	27 (11.4)	10 (11.9)	
Aspartate aminotransferase, IU/L	41.1+-24.4	41.2+-30.4	0.974
Creatinine, umol/L	73.6+-15.5	73.9+-16.3	0.875
neutrophil, 10 9/L	3.3+-1.5	3.5+-1.6	0.456
lymphocyte, 10 9/L	1.8+-0.7′	1.9+-0.8	0.321
**Pathology**			
Cirrhosis			0.941
Yes	195 (83.0)	70 (83.3)	
No	40 (17.0)	14 (16.7)	
Tumor factors			
Vascular invasion			0.708
No	110 (46.8)	35 (41.7)	
Microvascular invasion	98 (41.7)	39 (46.4)	
Macrovascular invasion	27 (11.5)	10 (11.9)	
Tumor diameter, cm	6.5+-4.0	6.6+-4.2	0.881
Tumor number			0.375
single nodule	139 (59.1)	45 (53.6)	
Multiple nodules	96 (40.9)	39 (46.4)	
Capsule			0.905
Complete	186 (79.1)	67 (79.8)	
Incomplete	49 (20.9)	17 (20.2)	
Edmonson-Steiner classification			0.636
I/II	144 (61.3)	54 (64.3)	
III/IV	91 (38.7)	30 (35.7)	
lymph nodemetastasis			0.442
Yes	7 (3.0)	4 (4.8)	
No	228 (97.0)	80 (95.2)	
Surgical factors			
Portal vein tumor thrombus			0.719
Yes	22 (9.4)	9 (10.7)	
No	213 (90.6)	75 (89.3)	
Clamping time, min			0.549
Yes	103 (43.8)	44 (52.4)	
No	132 (56.2)	40 (47.6)	
Blood transfusion			0.69
Yes	32 (13.6)	10 (11.9)	
No	203 (86.4)	74 (88.1)	
**Pre-pTACE**			
Alphae-fetoprotein, ng/mL			0.762
<200	191 (81.3)	67 (79.8)	
>=200	44 (18.7)	17 (20.2)	
Child-Pugh stage			0.362
A	199 (83.8)	74 (88.1)	
B	38 (16.2)	10 (11.9)	
HBV-DNA level, IU/mL			0.656
<=10 4	156 (66.4)	58 (69.0)	
>10 4	79 (33.6)	26 (31.0)	
Aspartate aminotransferase, IU/L	49.0+-40.2	50.1+-42.9	0.834
Creatinine, umol/L	69.4+-17.2	71.3+-21.2	0.396
neutrophil, 10 9/L	3.8+-6.7	4.7+-10.9	0.383
lymphocyte, 10 9/L	1.5+-0.7	1.5+-0.6	0.51
Platelets, 10 9/L	146.3+-60.1	139.6+-50.3	0.36

### Overall survival in the derivation and validation sets

**Figure 2:**
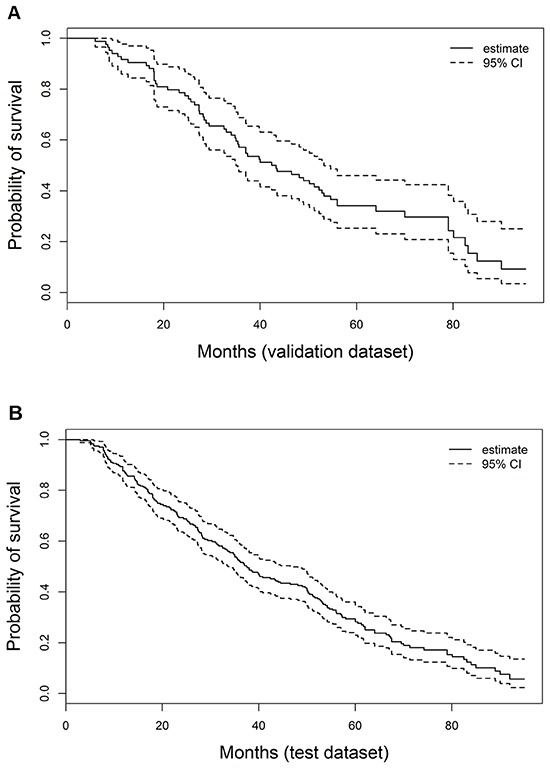
Kaplan-Meier estimates of HCC survival, respectively, in the derivation **A.** and validation **B.** sets.

As shown in Figure 2(a, b), the median follow-up was 37 months (range, 3 to 95 months) for the derivation set and 42.9 months (range, 5.8 to 95.0 months) for the validation set. In the derivation set, for patients with PA-TACE, the median OS was 37.4 months (inter-quartile range 19.0-64.0), the 1-, 3-, and 5-year OS rates were 87.7%, 52.1%, and 34.1%, respectively. In the validation set, for patients with PA-TACE, the median OS was 42.9 months (inter-quartile range 26.0-79.0), the 1-, 3-, and 5-year OS rates were 91.7%, 57.1%, and 34.1%, respectively.

### Development of prognostic nomogram

Baseline demographics were used for univariate analysis (Table 2). Seven risk factors provided a significant influence on prognosis, prior hepatectomy: BCLC stage, and tumor factor: vascular invasion, tumor diameter, and before PA-TACE: platelet count, HBV-DNA level, AFP level and Child-Pugh score change. These seven risk factors were accepted in the multivariate Cox regression analysis. After a stepwise removal of variables, five risk factors remained significant predictive value of prognosis (Table 3): HBV-DNA level prior PA-TACE procedure, platelet count, presence of vascular invasion, tumor diameter and Child-Pugh increase by 2 points or more.

**Table 2:** Univariate analysis results for prognostic factors in the derivation set

Variable		n=235	Overall Survival (months)		P-value (log rank)
			Median	95% CI	
Age	<65	211	37	32.4-41.6	
	>=65	24	44	27.7-59.3	0.894
Gender	Male	204	37	30.4-43.6	
	Female	31	38	30.1-45.9	0.678
Child-Pugh increase	Absent	67	44	28.5-59.5	
	+1 point	34	36.2	19.3-53.1	
	>=2 points	134	35	26.2-43.8	**0.015**
HBeAg	Positive	168	36	30.6-41.4	
	Negative	67	38	31.5-44.5	0.425
*HBV-DNA level (IU/mL)	<=10 4	156	48.1	40.1-56.1	
	>10 4	79	27.5	24.4-30.6	**<0.001**
BCLC stage	A	65	54.2	43.8-64.6	
	B	143	35	28.2-41.8	
	C	27	20.8	7.2-34.4	**<0.001**
AST Increase >25%	Absent	126	38	28.9-47.1	
	Present	109	35.6	25.8-45.4	0.202
*AFP (ng/mL)	<200	191	43.3	35.1-51.5	
	>=200	44	23.2	17.8-28.6	**<0.001**
*Cirrhosis	Absent	40	37	15.5-58.5	
	Present	195	37.4	31.0-43.8	0.452
*Vascular invasion	Absent	110	51.6	44.2-59.0	
	Microvascular invasion	98	31.4	26.2-36.6	
	Macrovascular invasion	27	18.3	10.8-25.8	**<0.001**
Tumor diameter (cm)	<5	96	54.2	41.3-67.1	
	>=5	139	31.2	25.3-37.1	**<0.001**
Tumor number (n)	1	139	43.3	32.8-53.8	
	>=2	96	33	26.4-39.6	0.095
Capsule	Complete	93	38.7	26.3-51.1	
	Incomplete	142	37	30.0-44.1	0.607
Edmonson-Steiner classification	I/II	144	39.8	29.4-50.2	
	III/IV	91	35	27.0-43.0	0.096
lymph nodemetastasis	Absent	228	39.2	31.6-43.7	
	Present	7	9.1	0-31.3	0.24
Clamping time	Absent	103	43.5	32.0-55.0	
	Present	132	35	30.1-40.0	0.234
Blood transfusion	Absent	210	35.1	29.3-44.6	
	Present	25	39.6	25.2-51.8	0.778
change of N/L	Increase	100	35.4	28.8-42.0	
	Decrease	135	42.4	31.2-53.6	0.058
*Platelets (10 9/L)	<100	178	34.7	29.5-39.9	
	>=100	57	54.2	46.4-62.0	**0.041**

**Table 3:** Multivariate stepwise backward Cox regression analysis for prognostic factors in the derivation set

Variable	Overall Survival		P-value (Cox)
	Hazard Ratio	95% CI	
Child-Pugh increase	1.264	1.063-1.502	0.008
HBV-DNA level (IU/mL)	1.612	1.179-2.205	0.003
Tumor diameter (cm)	1.67	1.101-2.534	0.016
Vascular invasion	1.36	1.029-1.798	0.031
Platelets (10 9/L)	0.659	0.461-0.941	0.022

**Figure 3:**
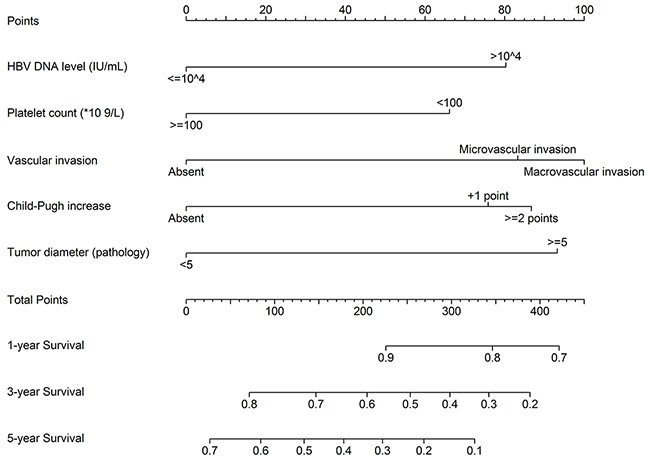
Nomograms for predicting HCC-specific survival probability after PA-TACE (1, 3 and 5 years)

**Figure 4:**
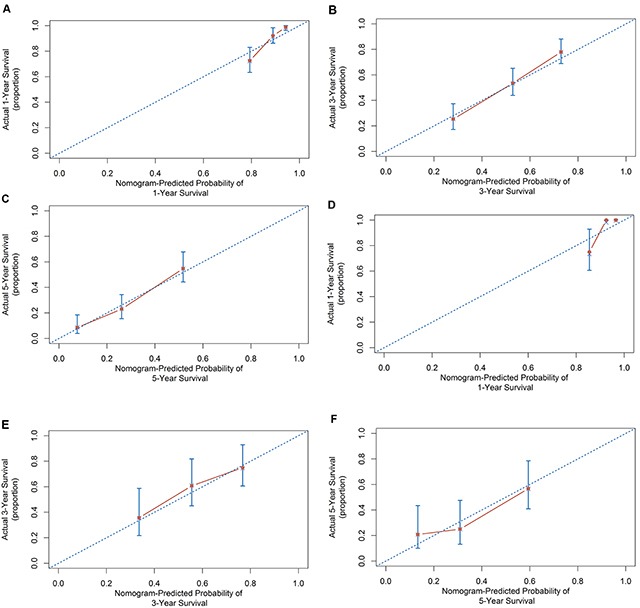
Calibration curve for predicting patient survival at **A.** 1 years, **B.** 3 years. **C.** 5 years in the derivation set and at **D.** 1 year, **E.** 3 years and **F.** 5 years in the external validation set.

These significant independently risk factors were integrated to form overall survival estimation nomogram in the derivation set (Figure 3). The nomogram demonstrated good accuracy for overall survival prediction, with a C index of 0.75 (95% CI, 0.67-0.83). The calibration plot for probability of survival at 1, 3, 5 year after TACE showed a fair agreement between the prediction by nomogram and actual observation (Figure 4a, 4b and 4c).

### Validation of prognostic nomogram

For the purpose of externally validating this model, we collected data among a second set of patients (n=84) undergoing PA-TACE at the Cancer Center of Sun Yat-sen University (Table 1). In this validation set, the c-index of the nomogram for predicting OS was 0.69 (95% CI, 0.56 to 0.83). The calibration plot for probability of OS at 1, 3, 5 year after PA-TACE showed a fair agreement between the prediction by nomogram and actual observation (Figure 4d, 4e and 4f).

### Risk of prognosis based on the nomogram scores

The optimal cutoff value of the total nomogram scores was determined to be 180. The sensitivity, specificity, positive predictive value, and negative predictive value when evaluated the prognosis risk of PA-TACE based on the nomogram were 76.4%, 65.9%, 90.7%, and 39.2% in the derivation set, and 78.1%, 60.0%, 86.2%, and 46.2% in the validation set, respectively (Table 4).

**Table 4:** Accuracy of the prediction score of the nomogram for estimating the prognosis risk of PA-TACE

Variable	Value (95% CI)	
	Derivation Set	Validation Set
Area under ROC curve	0.750 (0.672-0.827)	0.693 (0.559-0.826)
Cutoff score	180	180
Sensitivity, %	0.764 (0.698-0.823)	0.781 (0.660-0.875)
Specificity, %	0.659 (0.501-0.795)	0.6 (0.361-0.809)
Positive predictive value, %	0.907 (0.835-0.932)	0.862 (0.701-0.924)
Negative predictive value, %	0.392 (0.314-0.564)	0.462 (0.318-0.707)
Positive likelihood ratio	2.242 (1.476-3.407)	1.953 (1.124-3.393)
Negative likelihood ratio	0.357 (0.256-0.498)	0.365 (0.203-0.655)

## DISCUSSION

It is still controversial as to which kind of patients can benefit from the PA-TACE, because of the heterogeneity of the patients covered in the various studies, the clinical elements influencing prognosis importance were quite diverse and have some limitations, while there has been no reliable system to predict long-term prognosis. On the basis of our series of patients who had undergone hepatectomy combined with PA-TACE for HCC, we have created statistically predictive nomograms based on predictive Cox regression model tailored to the individual patient and give accurate prognostic information in these patients. The model is simple and easy-to-use, intergrating five predictors that make up the essentials of presurgical baseline characteristics, pathological findings of tumor, surgical operation factors and clinical evaluation prior to PA-TACE. The predictive performance of the model was further certified by external validation set. The statistical model provides a basis for clinicians and patients to select appropriate therapy after radical hepatectomy for HCC.

Recent studies have demonstrated the deterioration of liver function (defined as an increase of Child-Pugh score) before and after radical hepatectomy is known to be an importantly risk factor of worse prognosis. A study from Korean has developed clinical predictive nomograms recently in a large cohort of HCC patients undergoing surgical resection [8]. They found that liver function was an independent risk factor of OS identified by multivariate analyses (HR: 0.537, 95% CI, 0.434–0.665; P<0.001) and the result was confirmed by the two validation cohorts. On the other hand, because most patients with HCC suffer from liver cirrhosis, and surgical post-operative liver function could not be fully recovered in a relatively short time, TACE may aggravate deterioration of liver function and bring a worsen prognosis. Sieghart et al. [22] analysed the variation of the data before the first and second TACE in two sets, Child-Pugh score change was considered as a significant predictor of overall survival (HR: 4.4, 95% CI, 2.0–9.6; P<0.001). Likewise, the result is similar to those obtained in another study in France (HR: 3.03, 95% CI, 1.62–5.65; P=0.0005) [16]. In keeping with previous findings, the Child-Pugh score increase reflecting liver dysfunction is included in our proposed model.

A high HBV viral load is known to be a major risk factor for the development of HCC in patients with chronic HBV infection and for HCC recurrence after resection. Huang et al. [14] conducted a large comparative study of 1609 HCC patients with different serum HBV-DNA level. They concluded that there was significant relationship between HBV reactivation and HCC recurrence after partial hepatectomy, and postoperative high HBV-DNA level (>=200 IU/mL) was associated with a high HCC recurrence rate. Likewise, a Taiwanese cohort study conducted by Wu et al. [12] confirmed that high viral loads (HBV-DNA levels >10^6^ copies/ml) and hepatic inflammatory activity was correlated with the late recurrence in hepatitis B-related HCC patients. As expected, it is a crucial variable in our nomogram system.

The presence of microvascular invasion (MVI) is a histopathologic feature that indicates aggressive behavior of the HCC, which is a powerful validated risk factor of tumor recurrence and overall survival following surgical treatment. Currently, the diagnosis of MVI can only reliably be determined by pathologic histology of explanted tissue. Shim et al [10] have proposed a prognostic nomogram for prediction of recurrence and survival after HCC resection. Their results showed that MVI had high relative importance in recurrence-free survival (HR: 1.54, 95% CI, 1.21–1.95; P<0.001) and HCC-Specific Survival (HR: 1.71, 95% CI, 1.26–2.31; P=0.001) on the basis of the Cox model. Similarly, a cohort study conducted by Singapore medical center [23] also confirmed that MVI is a strong indicator of intrahepatic metastasis in HCC, which is a better predictor of tumor recurrence and long-term prognosis following surgical resection for HCC (HR: 2.12, 95% CI, 1.52–2.97; P<0.001). Our result is consistent with previous findings showing that the indicator in nomogram system is of great importance for prognoses.

Tumor diameter is a predictive covariate related to long-term prognosis in our models. Compared with patients only after hepatectomy, Sun et al. performed a cohort study involving 322 patients to assess the effectiveness of PA-TACE for HCC patients with MVI [23]. The maximum tumor diameter and PA-TACE were deemed as independently risk factors for both RFS and OS. However, the study has not further analysed what kind of patient groups is suitable for PA-TACE, and it also has not built related model based on the multivariable regression results. Our model provided a more comprehensive and powerful standard and basis for predicting prognosis of PA-TACE in HBV–related HCC. In addition, the low platelet count was noted to be a dependently risk indicator for PA-TACE in our study. Because cirrhosis have been confirmed in most of patients, together with hypersplenism/gastroesophageal varices. Recent study has suggested that a low platelet count played a significant role in microvascular invasion [24]. Meanwhile, the low platelet count before liver resection strongly linked with tumor recurrence and overall survival [10].

Our study provides some new insights and guidance for answering which patients with HBV–related HCC can receive the survival benefit from the PA-TACE. Above all, if patients exist with poor liver function and/or viral replication active after hepatectomy, PA-TACE can increase liver burden and worsen liver function. Meanwhile, the continuous high viral load after hepatectomy induce chronic inflammation in the liver remnant and may have impaired tumor immune surveillance and are more likely to develop multicentric carcinogenesis in the liver remnant [25]. It is finally significantly associated with tumor recurrence after hepatectomy. Protecting liver function and antiviral treatment prior PA-TACE not only effectively improves liver function, but also decreases the chance of developing a second primary HCC, which generates a better prognosis in HBV–related HCC patients. In addition, vascular invasion and larger tumor diameter significantly affect prognosis in HCC patients. Vascular invasion shows an aggressive tumor behavior and is closely linked to large tumor burden [26]. Moreover, patients with vascular invasion indicate a high frequency of fatal recurrence, multiple intrahepatic tumors and extrahepatic metastasis [27]. In theory, since HCC is supplied with rich blood flow, treatment with PA-TACE could kill or decrease residual tumor cell, thus eliminate micro-metastases in some extent and improve long-term survival outcome. Our study suggested HCC patients with vascular invasion and larger tumor diameter are more important and stronger risk factors for predicting prognosis, although PA-TACE could partly improve survival benefit. Thus, single interventional treatment could not completely prevent tumor recurrence. To confirm whether multiple preventive treatments and more closely follow-up are necessary, more high-quality, large-sample and multi-center randomized controlled trials are required, especially those recruit patients at different BCLC stages.

The major limitation of this study is that our data were acquired retrospectively and the population was restricted to HBV–related HCC. They could not be generalizable to prognostic prediction in all patients with HCC etiology other than HBV. It will certainly be necessary to futher verify our results among patients with HCC of various etiologies. Second, a prospective study is required to further confirm the reliability of the nomogram. Third, despite our nomogram shows a fairly good predictive accuracy for PA-TACE prognosis, with a cutoff point of 180, it had 15.0% and 45.0% false-positive and false-negative rates in the validation set, and 8.0% and 14.0% in the validation set, respectively.

## CONCLUSIONS

By combining five risk factors of PA-TACE, a novel, validated and widely applicable nomogram was constructed for predicting the prognosis of PA-TACE in patients with HBV-related HCC. The model should be conveniently used to facilitate the preoperative individualized prediction of PA-TACE in HBV-related HCC. It is warranted that the nomogram should be tested in prospective clinical trials.

## MATERIALS AND METHODS

### Patients

Consecutive patients, more than 18 years old at the time of the hepatic resection, diagnosed with HBV-related HCC by histopathology or radiological imaging (CT/MRI scans) according to European Association for the Study of the Liver (EASL) criteria [20] who underwent curative hepatectomy in the department of Hepatology and PA-TACE in the Interventional Radiology at the Affiliated Zhongshan Hospital of Fudan University between January 2005 and December 2009 (n=530) were screened for eligibility. These patients formed the derivation set of this study. From January 2006 to December 2008, another cohort of 84 patients treated in Sun Yat-sen University Cancer Center by PA-TACE after hepatectomy with the same selection criteria was analyzed as an independent external validation set.

Patients with HBV-related HCC at BCLC-stage A, B or C, and pre-surgical liver function status (Child-Pugh stage A or selected B) who received PA-TACE after hepatic resection within four weeks were included and formed the initial set for all further analysis.

Patients were excluded, if they received liver transplantation, or previous treatment for HCC. Additionally, patients who received hepatic resection despite poor liver function (Child-Pugh C), and blood tests were negative for hepatitis B surface antigen (HBsAg) or seropositive with one or more of the human immunodeficiency virus, HCV, or hepatitis D virus were ruled out.

All the patients were rechecked in our center 4 weeks after resection. If no recurrence was found, the PA-TACE treatment strategy was recommended. If the patients were found to have single/multiple tumors during the first evaluation 4 weeks after hepatectomy, they were regarded as tumor recurrence and excluded from this study.

Ethical approval for study protocol was provided by the Institutional review board of the Zhongshan Hospital and Sun Yat-sen University Cancer Center, and informed consent was obtained from all patients for their data to be used for research.

### Collection of data

Routine pre-surgical imaging (chest X-ray, abdominal ultrasound, liver protocol dynamic contrast-enhanced CT and/or MRI, chest computed tomography, and bone scans) was performed 5-7 days before the liver resection.

All laboratory values, including alpha-fetoprotein (AFP) as well as liver and renal function parameters including the Child-Pugh score, were determined one day before the hepatectomy and one day before the PA-TACE session. Viral tests, including hepatitis B surface antigen (HBsAg), hepatitis B e antigen (HBeAg) and hepatitis B virus deoxyribonucleic acid (HBV-DNA) load, were performed.

Additionally, the dynamic change in the Child-Pugh score (here after referred to as Child-Pugh score increase) between the time points pre-hepatectomy and pre-PA-TACE was recorded. All other time course change in liver/renal function-related parameters and neutrophil-to-lymphocyte ratio (NLR) between pre-hepatectomy and pre-PA-TACE were performed as outlined in the study design and statistical analyses section.

The histopathological evaluations of the resected specimens, including tumor number/diameter, degree of cirrhosis, microvascular invasion, presence of capsule/infringing capsule, differentiation of tumor cells (Edmonson-Steiner classification) and presence of portal vein tumor thrombus, were recorded by experienced pathologists as well as intraoperative blood/loss transfusion and portal clamping time.

All patients received regular evaluations, including serum biochemistry, liver function test, level of AFP value, and contrast-enhanced dynamic CT/MRI, every 3-4 months after PA-TACE until death or dropout from the follow-up program.

Recurrence was diagnosed based on the combined findings by measurement of their serum AFP level and CT/MRI scan. When recurrent tumor was confirmed during the study phase, the patients were actively treated with percutaneous ethanol injection, radiofrequency ablation, repeat liver resection, or TACE, according to the liver function status, tumor number and location HCC recurrence.

### Treatment procedures

#### Surgical procedure

Surgery was performed through a right bilateral subcostal incision. If necessary, the incision was extended to the left subcostal region. Surgeons carefully searched the abdominal cavity for the extent of local disease, extrahepatic metastases, and peritoneal seeding. The corresponding hepatic pedicle, hepatic vein, and short hepatic veins were ligated and divided. The size and number of the lesions and the relationship of the tumor to vascular structures were assessed by intrasurgical ultrasonography. Pringle's maneuver was applied to occlude the blood inflow of the liver with cycles of 15 minutes clamp time/5 minutes unclamped time. Liver resection was carried out using a clamp-crushing method [21]. Major/minor hepatic resection has been used in all surgery. Major hepatectomy was defined as resection of 4 or more liver segments. Minor hepatectomy was defined as resection of 3 or less liver segments.

### PA-TACE

When the liver function of the patient had recovered at 4 weeks after resection, TACE procedure was performed for the remnant liver. Angiography of celiac, hepatic, superior mesenteric, left gastric, and bilateral inferior phrenic arteries was performed using a 4F/5F catheter to identify all feeding arteries of any obvious tumor stains in the remnant liver using the Seldinger technique. An emulsion of 2-10 mL of Lipiodol Ultra-Fluide (Guerbet, France) mixed with 30-50 mg of EADM (Pfizer, USA) was then infused through a microcatheter (Progreat, TERUMO, Japan). The dosages of the chemotherapy drugs and lipiodol depended on the underlying state of liver function and body surface area [14]. The criteria for liver treatment used in both institutions were similar.

### Study design and statistical analyses

Numeric data are expressed as means and SDs, and categorical data are shown as frequency and proportion.

Patient's characteristics in the derivation and validation set are presented with descriptive statistics. OS was defined as the time from the PA-TACE until death or last follow-up. Survival curves were calculated using the Kaplan-Meier method; median overall survival and their 95% confidence intervals are reported. Univariate analysis of the OS was performed on the derivation set. Log-rank test was performed to detect significant parameters in univariate analysis. Variables that were significantly associated with survival in the univariate analysis (P<0.05) entered a stepwise Cox regression model (conditional backward selection). Multivariate Cox regression analysis with stepwise selection was used to detect independent predictors used in a nomogram. The nomogram was formulated using the rms package in R version 3.2.0.

The predictive performance of the nomograms was measured using the concordance index (C-index) and plotting the Kaplan–Meier curves of the quartiles of predictions, and was illustrated by drawing calibration plots. Model validation was performed using bootstraps with 1000 resamples to quantify the overfitting of modeling strategy and predict future performance of the model. Statistical analyses were performed using the R software version 3.2.0 (http://www.rproject.org/). All statistical tests were two-tailed and a p value<0.05 was considered statistically significant.
